# Automating Detection of Drug-Related Harms on Social Media: Machine Learning Framework

**DOI:** 10.2196/43630

**Published:** 2023-09-19

**Authors:** Andrew Fisher, Matthew Maclaren Young, Doris Payer, Karen Pacheco, Chad Dubeau, Vijay Mago

**Affiliations:** 1 Department of Mathematics and Computing Science Saint Mary's University Halifax, NS Canada; 2 Canadian Centre on Substance Use and Addiction Ottawa, ON Canada; 3 Greo Evidence Insights Guelph, ON Canada; 4 Department of Psychology Carleton University Ottawa, ON Canada; 5 Canadian Medical Protective Association Ottawa, ON Canada; 6 Faculty of Health York University Toronto, ON Canada

**Keywords:** early warning system, social media, law enforcement, public health, new psychoactive substances, development, drug, public health, dosage, Canada, Twitter, development, poisoning, monitoring, community, public safety, machine learning, Fleiss, tweet, tweet annotations, pharmacology, addiction

## Abstract

**Background:**

A hallmark of unregulated drug markets is their unpredictability and constant evolution with newly introduced substances. People who use drugs and the public health workforce are often unaware of the appearance of new drugs on the unregulated market and their type, safe dosage, and potential adverse effects. This increases risks to people who use drugs, including the risk of unknown consumption and unintentional drug poisoning. Early warning systems (EWSs) can help monitor the landscape of emerging drugs in a given community by collecting and tracking up-to-date information and determining trends. However, there are currently few ways to systematically monitor the appearance and harms of new drugs on the unregulated market in Canada.

**Objective:**

The goal of this work is to examine how artificial intelligence can assist in identifying patterns of drug-related risks and harms, by monitoring the social media activity of public health and law enforcement groups. This information is beneficial in the form of an EWS as it can be used to identify new and emerging drug trends in various communities.

**Methods:**

To collect data for this study, 145 relevant Twitter accounts throughout Quebec (n=33), Ontario (n=78), and British Columbia (n=34) were manually identified. Tweets posted between August 23 and December 21, 2021, were collected via the application programming interface developed by Twitter for a total of 40,393 tweets. Next, subject matter experts (1) developed keyword filters that reduced the data set to 3746 tweets and (2) manually identified relevant tweets for monitoring and early warning efforts for a total of 464 tweets. Using this information, a zero-shot classifier was applied to tweets from step 1 with a set of keep (drug arrest, drug discovery, and drug report) and not-keep (drug addiction support, public safety report, and others) labels to see how accurately it could extract the tweets identified in step 2.

**Results:**

When looking at the accuracy in identifying relevant posts, the system extracted a total of 584 tweets and had an overlap of 392 out of 477 (specificity of ~84.5%) with the subject matter experts. Conversely, the system identified a total of 3162 irrelevant tweets and had an overlap of 3090 (sensitivity of ~94.1%) with the subject matter experts.

**Conclusions:**

This study demonstrates the benefits of using artificial intelligence to assist in finding relevant tweets for an EWS. The results showed that it can be quite accurate in filtering out irrelevant information, which greatly reduces the amount of manual work required. Although the accuracy in retaining relevant information was observed to be lower, an analysis showed that the label definitions can impact the results significantly and would therefore be suitable for future work to refine. Nonetheless, the performance is promising and demonstrates the usefulness of artificial intelligence in this domain.

## Introduction

The rapid emergence of new psychoactive substances (NPSs) on the global drug market is a phenomenon that creates challenges for public health and drug policy alike [1,2]. Surveillance through the United Nations Office on Drugs and Crime (UNODC) early warning system (EWS) has detected over 1100 individual NPSs across 133 countries by the end of 2021, with North America showing some of the highest numbers [3]. While some of these substances are widely available and marketed, there is typically little to no information to consumers about their types, safe dosing, or potential adverse effects [3,4]. Moreover, NPSs are being consumed unintentionally as a result of the contaminated unregulated drug supply (particularly nonmedical benzodiazepines and synthetic opioids; Canadian Community Epidemiology Network on Drug Use reports or alerts), driving the record-high drug poisoning rates observed in North America and elsewhere [5].

The rapidly evolving and unregulated nature of this market poses a challenge in collecting and tracking up-to-date information regarding drug use harms. This can slow down policy interventions aimed at developing harm reduction and prevention practices [6,7]. The lack of information on emerging substances on the unregulated market, coupled with the rapid proliferation of reported harms, demonstrates a need for more systematic collection, analysis, interpretation, and dissemination of timely and accurate information on the availability, use, and harms associated with new drugs and trends, especially as deaths associated with fentanyl and psychostimulants are increasing [8,9] and NPSs continue to appear on the market [10].

These functions are accomplished through the development of EWSs. EWSs enable the systematic detection of new and emerging drug trends by monitoring specific data sources for relevant information and issuing alerts and public health notifications when warranted. These outputs can then guide the development of interventions that prevent and reduce associated harms. For this reason, EWSs are routinely used by public health, emergency response, and law enforcement bodies.

Several large international public health and law enforcement organizations issue such notifications (eg, UNODC, United Nations Commission on Narcotic Drugs, World Health Organization, European Monitoring Centre for Drugs and Drug Addiction, and CICAD’s Inter-American Observatory on Drugs), providing a rich source of drug monitoring information. However, smaller national or regional organizations (eg, National Drug Early Warning System and Canadian Community Epidemiology Network on Drug Use) are better positioned to collect detailed and timely information from local departments of public health and local law enforcement detachments. However, in Canada, there is a very large number of municipal public health units and local law enforcement detachments that occasionally communicate relevant information, making it difficult to monitor all relevant data sources for relevant information at all times.

In recent years, notifications have started to be communicated to the public through social media sources. Twitter in particular is a social media platform that has widespread use and is therefore actively used by local law enforcement and public health groups as a primary method of sharing information alerts, including drug-related warnings. This kind of information is then used by those working in public health and harm reduction, emergency medical response services, primary health care, and law enforcement to develop or adapt targeted responses to new drug-related health threats. That is, while the tweets are issued by local organizations to inform the local public, the aggregation and synthesis of those tweets across regions and time can serve as an early warning function to those relying on EWSs for their public health and safety mandates.

While monitoring local notifications on Twitter is a promising avenue, the entities issuing the tweets typically warn the public about a great number of public health threats (eg, salmonella food contamination and heat wave warnings), whereas the proportion of notifications specifically relevant to drug harms is relatively small. This makes monitoring, filtering, and mining these data sources for relevant information a challenging and arduous task. Combined with the large number of local organizations regularly issuing these kinds of alerts, the data landscape becomes extremely complex, making it difficult to detect important signals of drug-related harms.

Previous work has shown that social media can be harnessed to study health-related phenomena and monitor public health activities [11]. Recent studies have drawn from social media to examine trends in illicit drug use [12], prescription medication misuse [13], adverse drug reactions [14], and overdoses [15]. In addition, studies have used machine learning techniques to automate the process of identifying and screening relevant health content. For instance, machine learning has been used to detect and track influenza in the United Kingdom [16], identify vaping-relevant tweets and characterize associated sentiments [17], identify illegal web-based sale of prescription opioids [18], and track adverse drug reactions [19]. Another system [20] was developed to extract disease-related news from a fixed corpus to help researchers track epidemics. Together, the evidence shows that social media is a valuable data source that can be mined for important and current information. However, at present, this potentially rich source of information is not being filtered or systematically monitored for early detection of emerging drugs and ongoing associated harms.

Given the extremely time-consuming nature of manually scanning and extracting tweets regarding drug-related harms and trends, this proof-of-concept study sought to investigate the feasibility and use of a semisupervised algorithm to detect and collect these drug-related harms tweets. Specifically, the aim of this pilot study was to determine how reliably an automatic, machine learning algorithm can identify tweets relevant to NPS-related risks and harms, compared to manual selection, from a set of public health and enforcement tweets. Successful automation could set the foundation for EWSs to identify NPS trends and monitor ongoing drug-related harms, alert their stakeholders, and create more opportunities for local responses. As a result, to the best of our knowledge, the following contributions are made: (1) a framework that uses zero-shot classification for detection of drug-related harms; (2) a novel data set of filter words and classified Twitter posts by subject matter experts; and (3) an analysis of the drug-related tweets from law enforcement and public health agencies in Quebec, Ontario, and British Columbia in Canada.

## Methods

### Overview

The study consisted of the following steps: (1) compiling a raw data set of tweets and preparing for the analysis: preprocess the data set to remove unquestionably irrelevant tweets and have 3 subject matter experts (SMEs) review the remaining tweets manually, selecting relevant ones; and (2) train the algorithm to pick out relevant tweets and compare performance to the manual selection and labels. These are described in turn below.

### Data Collection

To develop the data set, 145 Twitter accounts related to law enforcement and public health agencies were manually selected in 3 Canadian provinces: Quebec (n=33), Ontario (n=78), and British Columbia (n=34). Next, access to an academic license for the Twitter application programming interface (API) was granted which allows for up to 10 million tweets per month to be retrieved at no cost to researchers. Next, a Python script was developed to collect daily tweets for each predefined username and append it to a comma-separated values file along with their respective time stamps. Once this process was completed over the span of August 23, 2021, to December 21, 2021, a total of 40,393 tweets were captured. From this, the first preprocessing step was to identify irrelevant tweets based on a set of remove and keep words where a tweet was removed if, and only if, it contained a remove word but no keep words. As an example, consider the following sets:

remove_words = (covid, pandemic, vaccine,...)

keep_words = (warrant, fentanyl, overdoses,...)

To elaborate further, assume the text of a tweet as, “Due to the pandemic, there has been an increase in overdoses,” which would initially be flagged for removal based on “pandemic” but would ultimately be kept as it contains “overdoses.” To do this, we consulted with three SMEs who have the following qualifications: (1) a senior knowledge broker with over 20 years of experience in communicating messages relevant to substance use risks and harms and coordination of knowledge exchange networks; (2) an information specialist working in the substance use and addiction field for 19 years, including managing Addiction News Daily (a media monitoring service) for the past 15 years; and (3) a substance use researcher with a keen interest in artificial intelligence (AI) applications and over 10 years of quantitative and qualitative experience within health care and biological sciences, including contributions to systematic reviews as an external coder to ensure consistency and interrater reliability.

The resulting lists contained a total of 206 (remove) and 26 (keep) words, respectively, that can be found in our repository [21]. Once this was applied to the raw data set, only 3746 tweets were kept (~9.3%) from the data collection process. Finally, to identify which of the social media posts were relevant for the study, 3 SMEs manually analyzed the contents of all 3700 tweets and labeled ones containing drug-related events such as drug seizures or drug alerts with a binary label to keep or not-keep.

### Machine Learning

From the data collection and labeling process, it can be observed that a very small portion was relevant to the human annotators for drug-related events. Therefore, it would be desirable to have an automated means of classifying each tweet such that its relevancy is determined in real time. To achieve this goal, our work uses a zero-shot classifier [22] that has been pretrained on several data sets across a variety of different languages [23]; the tweets in this study were in English as well as French. Since the published model is being used with its default configuration [22], there is no need to perform additional fine-tuning or parameter optimization. Additionally, due to the multilingual requirement, there were a limited number of models to choose from so the one with the highest performance based on a literature search was implemented [22]. In summary, the model is first given a set of predefined labels (eg, drug arrest). Then, for a given text input, it outputs a corresponding set of probabilistic values to show which one is the most likely match. After consulting with the SMEs and analyzing a previous publication [24], consider the following label set that was used in this work:

labels = (drug arrest, drug discovery, drug report, drug addiction support, public safety report, other)]

where tweets under the first 3 categories are kept, and ones under the last 3 are discarded. Since the goal of this project is to determine which are relevant for drug-related events, the accuracy of this binary decision [25] (ie, keep or not-keep) is evaluated rather than a per category accuracy.

### Computational Resources

Since the machine learning model used in this process has already been trained (through previous iterations), the requirements to run it are minimal. In particular, the main graphical processing unit used in this project was an NVIDIA Tesla K80 [26], which was released toward the end of 2014. Furthermore, since the data set size is relatively small, central processing unit and RAM requirements are negligible assuming that a modern-day computer is being used. For a given input, the zero-shot classifier [22] takes approximately 5 seconds to produce this final output. This means that the automatic analysis of our 3746 tweets (collected over the span of 4 months) will take approximately 5 hours (or approximately 2.5 min per day) to complete.

### Ethical Considerations

To compile the data set used in this study, publicly available information from health and law enforcement groups was collected via the Twitter API. Therefore, ethics approval was not required to report the analysis of public behaviors.

## Results

### Human Annotator Agreement

Once the 3 SMEs decided which tweets to keep or not-keep, a consensus set of labels was used to define the annotated data as a binary decision [25]. To quantify these results, an analysis was performed to determine the Fleiss κ score [27], which was found to be ~0.731, with 464 tweets marked as being relevant (~12.5% or 464/3707 tweets of the cleaned data set). As a result, the score shows that the SMEs have a moderate to strong agreement between one another [28]. This final data set was then used to compare against machine learning algorithm-generated labels.

### Machine Learning

**Figure 1:**
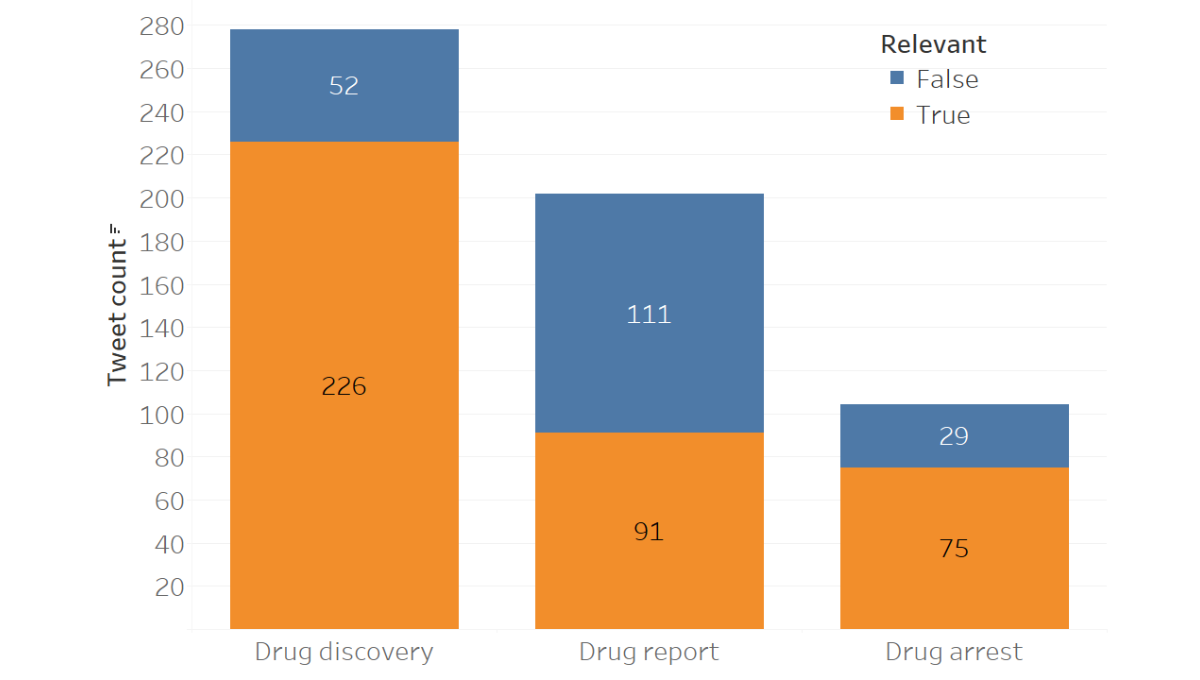
The distribution of keep tweets by the system (n=584), where orange denotes relevant tweets by the subject matter experts (n=392).

**Figure 2:**
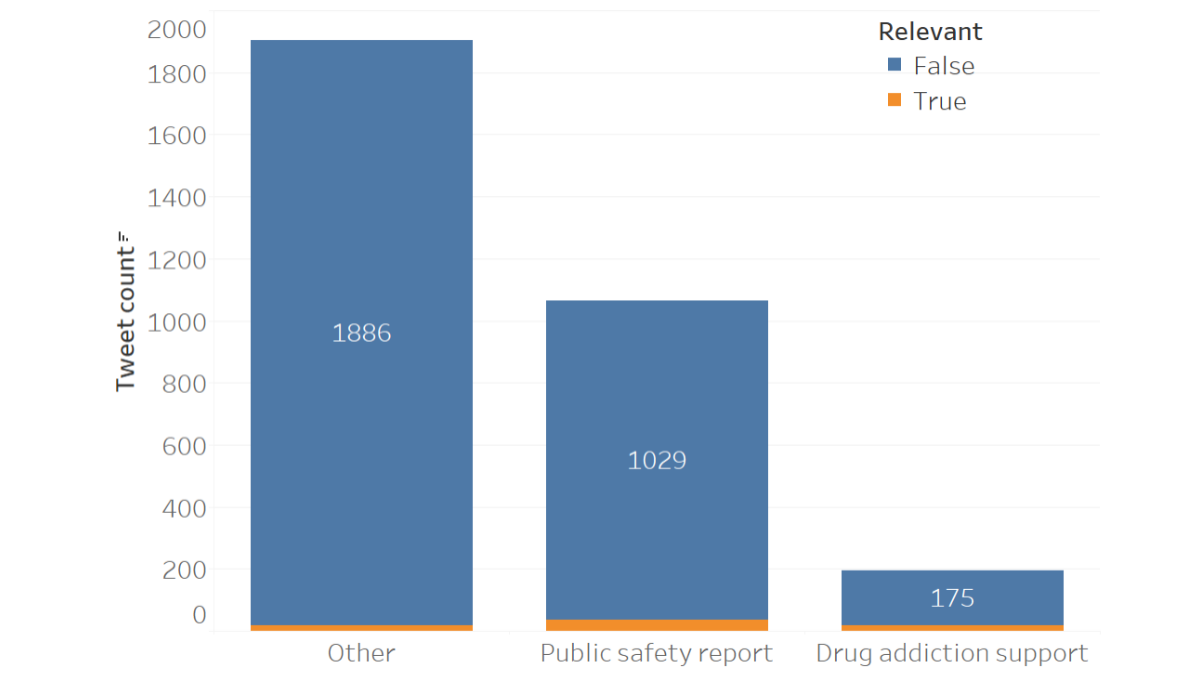
The distribution of not-keep tweets by the system (n=3162) where blue denotes irrelevant tweets (n=3090).

After applying the zero-shot classifier [22] to all 3707 tweets, the output was compared with the annotated data to see how much of an overlap could be observed. When looking at the keep label results (ie, drug discovery, drug report, and drug arrest), a total of 584 tweets were captured with an overlap of 392 (Figure 1). Since there were a total of 464 relevant tweet annotations, this gives the system an ~84.5% (392/464) accuracy in making this decision. Conversely, when looking at the not-keep label results (ie, drug addiction support, public safety report, and others), a total of 3162 tweets were captured with an overlap of 3090 (Figure 2). This demonstrates an accuracy of ~94.1% (3090/3282) when identifying irrelevant tweets presented to the system.

**Figure 3:**
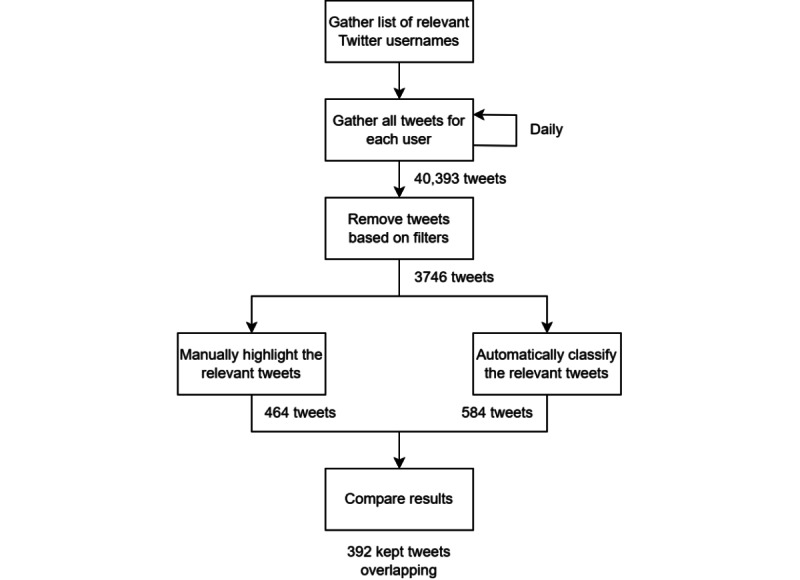
A summary of this work’s overall process.

Although the system is ultimately making a binary decision [25], it is important to analyze how much of an impact each label had on the results. For the keep labels, consider Table 1 which shows that “drug discovery” contributed significantly more to the ~84.5% (392/464) accuracy than the other 2. As for not-keep labels, consider Table 2 where it can be observed that “other” contributed the most to the ~94.1% (3090/3282) accuracy. This demonstrates the importance of accurately defining these sets as the contribution is not evenly distributed across each label [28]. To summarize how the comparison to the SMEs is performed, consider Figure 3 which shows the complete process from data collection to label comparison.

**Table 1:** The per-label accuracy contribution (total: 392/464, ~84.5%) for tweets kept by the system.

Label	Accuracy contribution, n (%)
Drug arrest	75 (~16.2)
Drug discovery	226 (~48.7)
Drug report	91 (~19.7)

**Table 2:** The per-label accuracy contribution (total: 3090/3282, ~94.1%) for tweets not kept by the system

Label	Accuracy contribution, n (%)
Drug addiction support	175 (~5.3)
Public safety report	1029 (~31.3)
Other	1886 (~57.4)

## Discussion

### Principal Findings

The results from this proof-of-concept study suggest that AI is a feasible mechanism for monitoring social media and detecting signals of drug-related risks and harms. While further refinement is needed, the system presented here can significantly reduce the manual labor of identifying signals of emerging drug threats and harms. This provides a first step toward automated data collection that can feed into EWSs and ultimately contribute to national monitoring and surveillance efforts.

### System Accuracy

Of the 3746 tweets in this study (narrowed down from the raw data set of 40,393), the SMEs classified 464 as relevant and 3282 as irrelevant, while the AI system classified 584 as relevant and 3162 as irrelevant. Assuming SME judgment as the gold standard, system performance can therefore be summarized as (1) 392 true positive (correctly classified as relevant), (2) 192 false positive (incorrectly classified as relevant), (3) 3090 true negative (correctly classified as irrelevant), and (4) 72 false negative (incorrectly classified as irrelevant). This gives the system ~94.1% (3090/3282) sensitivity and ~84.5% (392/464) specificity [28].

### System Sensitivity

In this context, sensitivity refers to the system’s ability to remove irrelevant tweets without accidentally or indiscriminately removing relevant tweets alongside. At ~94.1% (3090/3282), the system is sufficiently good at this; if it removes a tweet, it is very likely to be truly irrelevant. Figure 2 illustrates this as the large number of tweets classified as irrelevant by both the SMEs and the system (n=3090; blue) and the small number of tweets classified as relevant by the SMEs but irrelevant by the system (n=72; orange). The incorrect removal of 72 tweets suggests that the system may be overly sensitive and may remove tweets that actually contain relevant information, leading to a loss of signal. However, this number was small and was not systematic across the Twitter accounts, so the loss is negligible.

In the context of monitoring for drug-related harms, this means that automation would cause some, but not much, signal to be lost (ie, misclassified as noise), allowing for confidence that the set of keep tweets contains all the relevant information. However, this does not guarantee that it contains only the relevant information (see System Specificity below).

### System Specificity

In this context, specificity refers to the system’s ability to accurately distinguish relevant from irrelevant tweets and exclusively keep the relevant ones. At ~84.5% (392/464), the system is less efficient at this. Figure 1 illustrates that while it correctly kept most of the tweets classified as relevant by the SMEs (n=392; orange), it also kept a fair number of tweets classified as irrelevant by the SMEs that should have been removed (n=192; blue). This means that while the set of keep tweets contains all or most of the relevant signal, it also still contains noise.

In the context of monitoring for drug-related harms, this means that there would still be the need for manual review and decision-making among kept tweets, decreasing the usefulness of the automation as there is still the need to invest time and labor. Eventually, the goal would be to increase this accuracy to minimize the need for manually discarding irrelevant tweets. This could be achieved by developing another, larger manually labeled data set that could be used to fine-tune the machine learning model further.

### Implications

The results from this proof-of-concept study are promising for the future of monitoring social media for mentions of drug-related events. The capacity to identify clusters of harms related to substance use that may warrant further investigation would be highly complementary to existing national drug EWSs. Such event-based surveillance methods have been used to monitor infectious disease outbreaks [29].

The system presented in the study is effective at removing irrelevant tweets without removing relevant ones. However, manual review and decision-making are still required among the tweets kept by the system. Improving the specificity of the system in the future could minimize the need for manually discarding irrelevant tweets by using the methods proposed in references [30,31]. Developing a larger manually labeled data set could be used to fine-tune the machine learning model further and increase the accuracy of the system.

The results achieved through this automation can feed into EWSs and contribute a far more comprehensive and detailed new data set to national monitoring and surveillance efforts than what could be achieved through human resources alone. While it is difficult to calculate the time saved by this automation, the difference between the speed of the model (seconds) compared to that of the experts (hours) in categorizing tweets suggests that the time savings would be significant.

Overall, the results of this study demonstrate the potential for AI to play an important role in automated data collection and analysis for surveillance by public health and safety entities engaged in decreasing drug-related risks and harms.

### Regional Considerations

To further break down the filtered data set of 3746 tweets, consider Figure S1 in Multimedia Appendix 1, which shows the per-province counts. In comparison to the user count distribution (Quebec: n=33, 22.8%; Ontario: n=78, 53.8%; British Columbia: n=34, 23.4%), it can be observed that the tweet distribution is not quite proportionate as fewer were captured in Quebec. Similarly, when looking at tweets labeled as relevant by the SMEs in Figure S2 in Multimedia Appendix 1, the same effect in the distribution can be observed, where the majority were distributed in Ontario (n=354), followed by British Columbia (n=98) and Quebec (n=25). A breakdown of the tweets kept by the system per province has also been provided in Figures S3-S5 in Multimedia Appendix 1 to analyze the tweets from the top 10 users. For example, in Ontario (Figure S4 in Multimedia Appendix 1) all tweets kept by the system for user “GuelphPolice” had been labeled by the annotators as being relevant whereas for user “HKPRDHU,” 8 out of the 17 kept had been labeled as irrelevant. This information is important to consider as it could assist in determining whether or not a Twitter account should be monitored by the system.

### Limitations and Future Work

Since this study used a pretrained model, the accuracy of the results can only be improved by changing the labels or creating a large data set for fine-tuning. This would require a significant amount of additional labeling as preliminary results showed that just ~12.5% (464/3707) of the filtered data were found to be relevant. To assist in this effort, future works could use the presented results as a validation data set to ensure that their approach is aligned with the SMEs. A breakdown of the Twitter handles that contributed to the data set is shown in our repository [21] where it can be observed that a uniform distribution is spread across several accounts to provide a variety of different writing styles.

Once an EWS has been created, a web interface could be developed to provide those interested in substance use epidemiology and monitoring drug-related harms an accessible means of interpreting the data. This would display a real-time feed of relevant tweets along with their classification (ie, drug arrest, drug discovery, or drug report) to justify the results to the end user. If there is a disagreement with the labeled information, feedback could also be collected to help improve the accuracy of the model. Since the professional and public health organization accounts being monitored are providing health information and safety messaging as a public service, this system could increase their impact on the national effort to reduce drug-related harms.

### Conclusions

In this preliminary study, an automated system to capture tweets containing drug-related events was examined for feasibility and accuracy. To begin, a list of usernames was predefined and monitored through the Twitter API for 4 months, resulting in a raw data set of 40,393 tweets. Then, using filters created from consultation with subject matter experts, 3707 tweets were kept in the cleaned data set with 477 of them being manually labeled as containing drug-related events. From this, a zero-shot classifier was applied to make a keep or not-keep decision where the results showed an ~84.5% (392/464) and ~94.1% (3090/3282) accuracy, respectively. The system was successful in removing irrelevant tweets with ~94.1% (3090/3282) accuracy but achieved only ~84.5% (392/464) accuracy in its ability to exclusively keep relevant tweets. Nonetheless, since the number of relevant tweets is quite small overall (464/3707, ~12.5%), this performance is promising as it greatly reduces the manual effort that would otherwise be required to perform such a task. Future research should refine the model to improve its specificity by experimenting with the labels and should also examine a way to optimize subclassifications for the binary decision.

## Appendix

Multimedia Appendix 1 A breakdown of the tweet distribution per province, as well as for the top 10 users.

## Abbreviations

AI: artificial intelligence
API: application programming interface
EWS: early warning system
NPS: new psychoactive substance
UNODC: United Nations Office on Drugs and Crime

## Data Availability

The data sets generated and analyzed during this study are available in the CCSA Project repository [21].
