# Supplementary material for: Automating Detection of Drug-Related Harms on Social Media: Machine Learning Framework
Source: J Med Internet Res. 2023 Sep 19;25:e43630. doi: 10.2196/43630 (PMC10548323; doi:10.2196/43630)
Supplement: Multimedia Appendix 1 [file jmir_v25i1e43630_app1.docx]

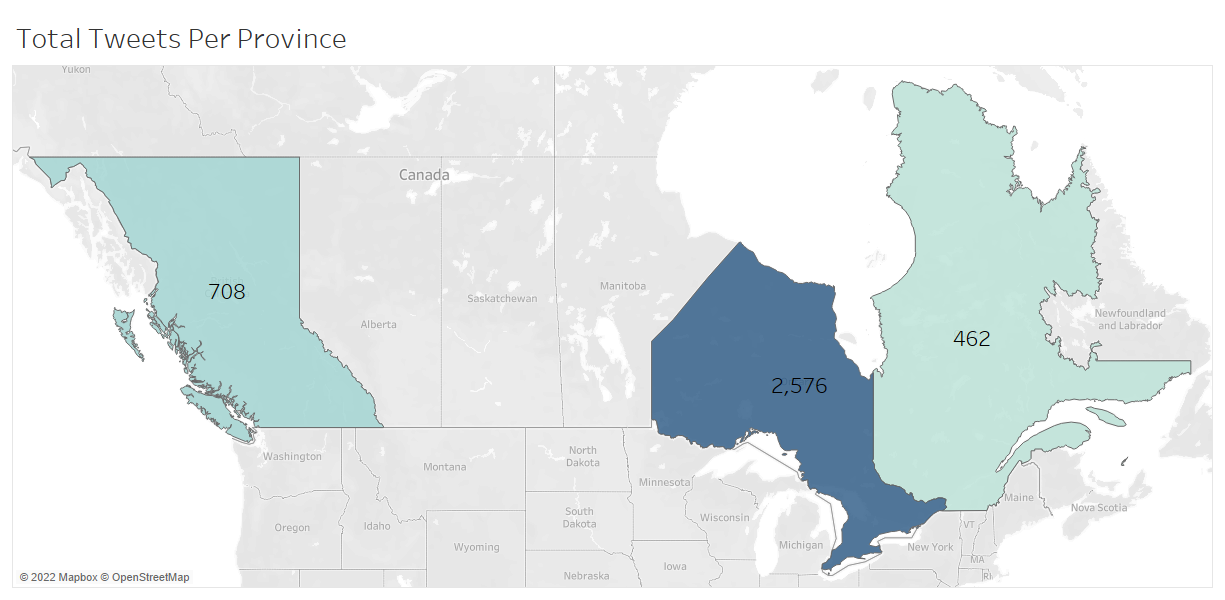


**Figure S1.** Distribution of all tweets per province.


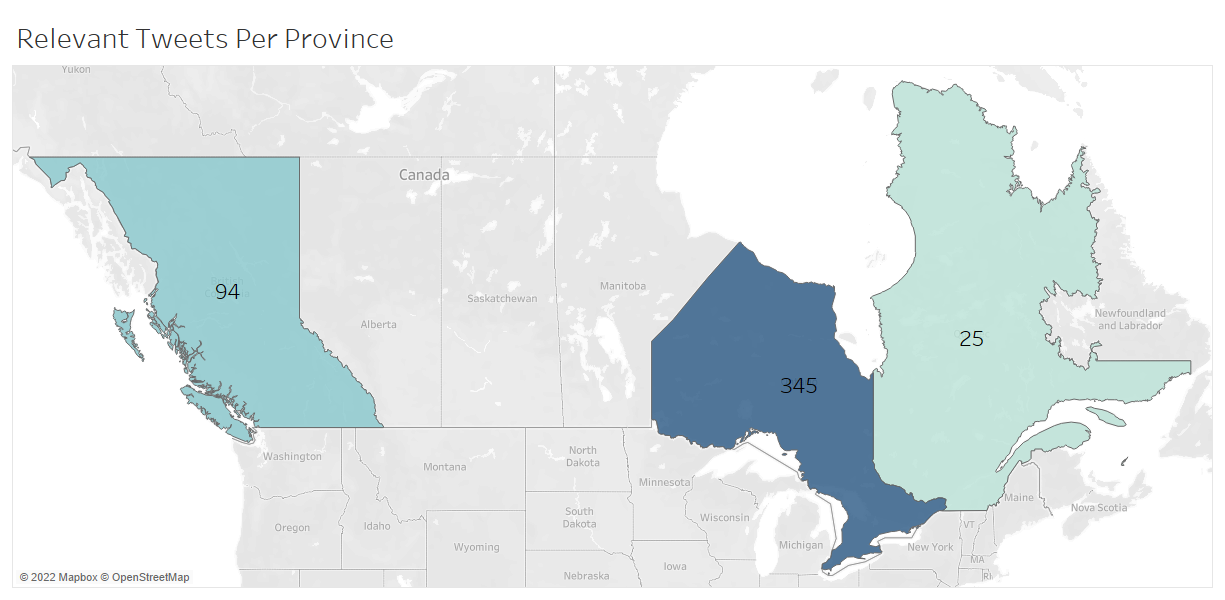


**Figure S2.** Distribution of relevant tweets per province.


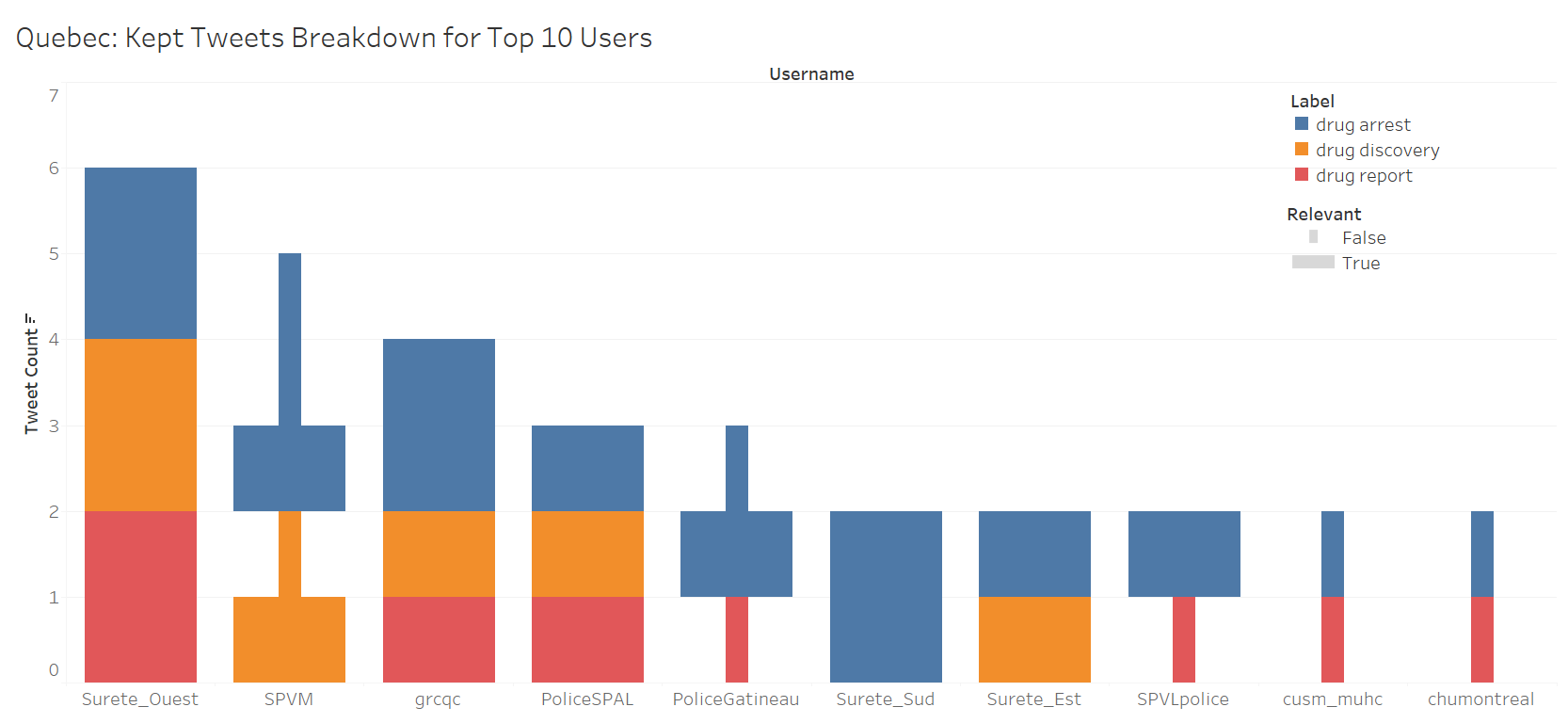


**Figure S3.** Tweet breakdown for the top 10 users in Quebec.


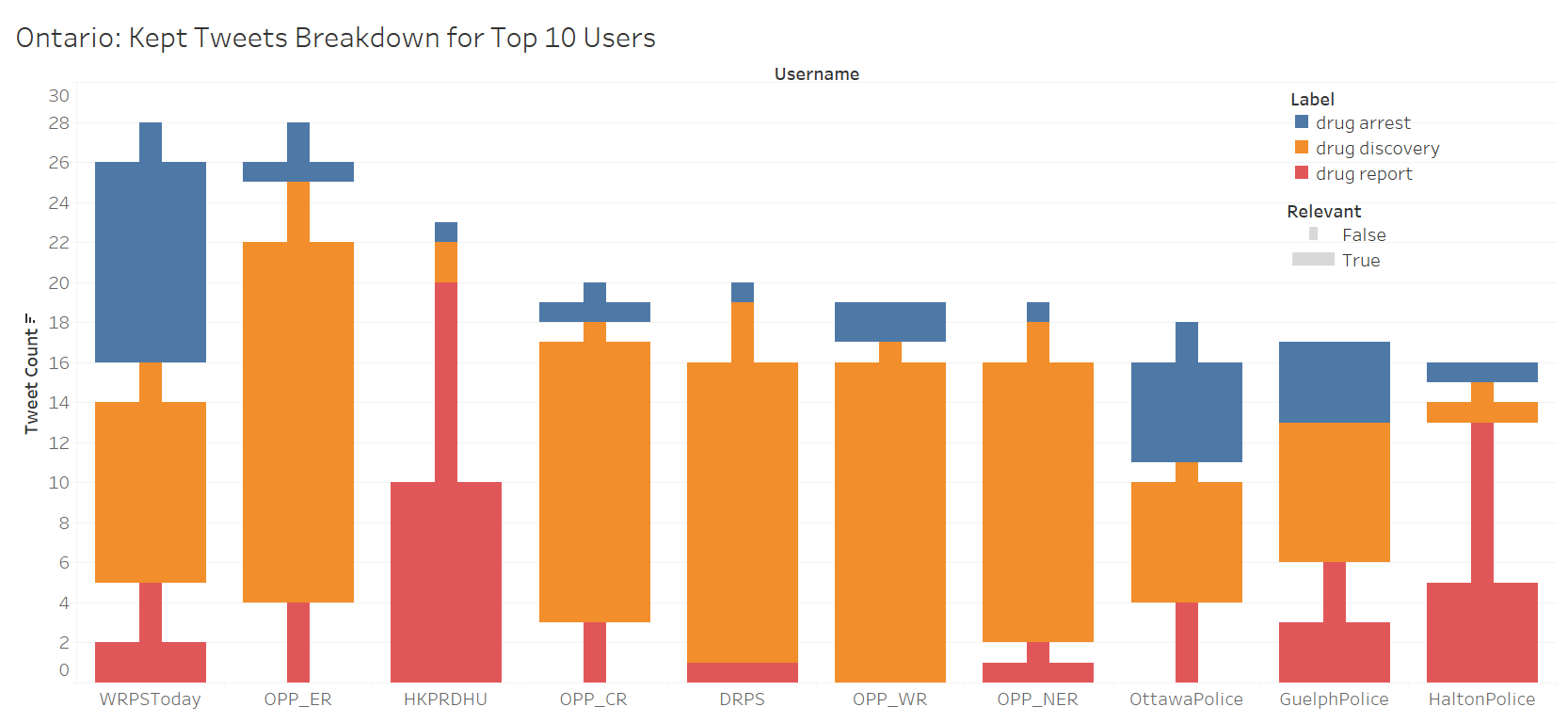


**Figure S4.** Tweet breakdown for the top 10 users in Ontario.


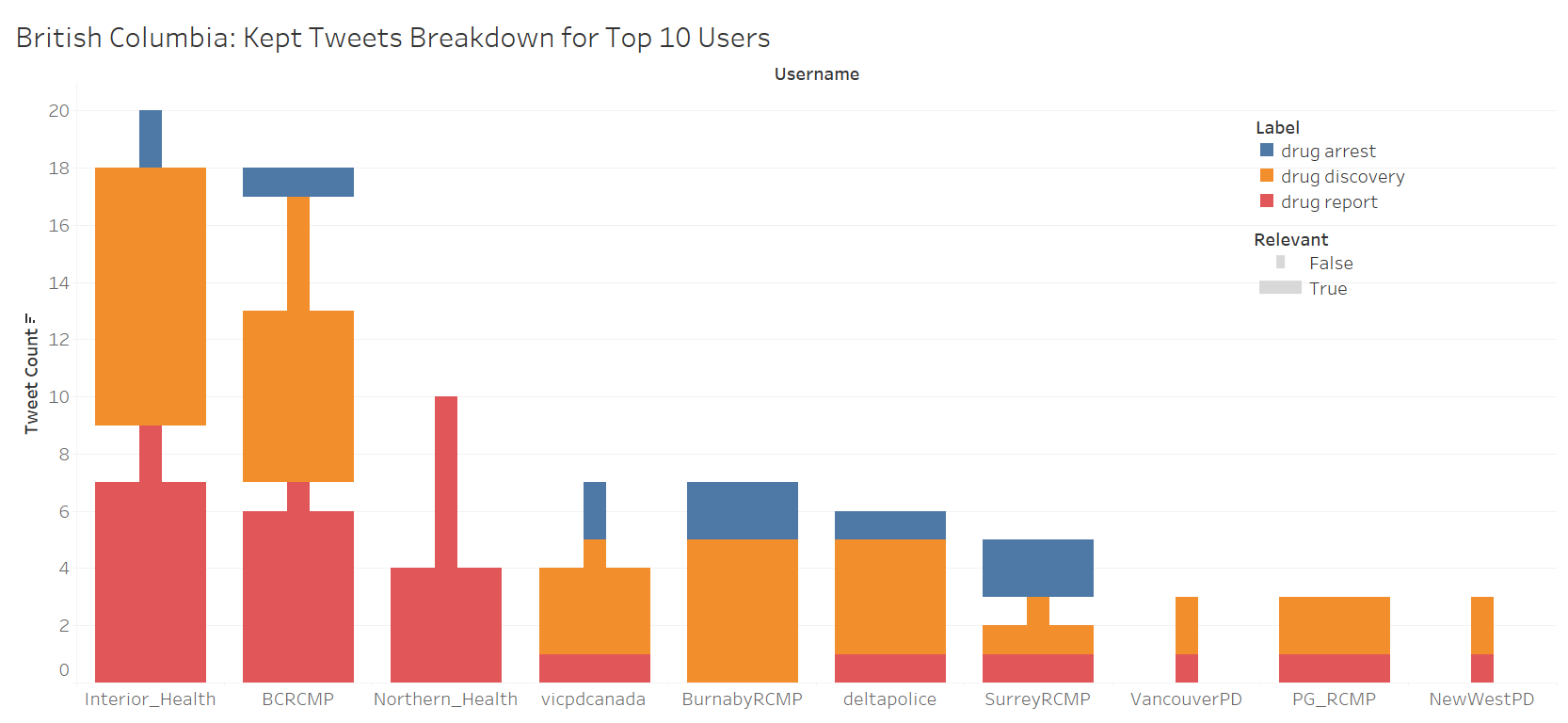


**Figure S5.** Tweet breakdown for the top 10 users in British Columbia.
